# Prognostic value of micro-RNA 375, 133, 143, 145 in esophageal carcinoma: A systematic review and meta-analysis

**DOI:** 10.3389/fonc.2022.828339

**Published:** 2022-09-13

**Authors:** Pinhao Fang, Jianfeng Zhou, Xiaokun Li, Siyuan Luan, Xin Xiao, Qixin Shang, Hanlu Zhang, Yushang Yang, Xiaoxi Zeng, Yong Yuan

**Affiliations:** ^1^ Department of Thoracic Surgery, West China Hospital, Sichuan University, Chengdu, China; ^2^ West China Biomedical Big Data Center, West China Hospital, Sichuan University, Chengdu, China

**Keywords:** micro-RNA, esophageal carcinoma, prognosis, meta-analysis, biomaker

## Abstract

Many studies have confirmed that micro-RNA (mir) is related to the prognosis of esophageal carcinoma (EC), suggesting the mir could be used to guide the therapeutic strategy of EC. Some of mir molecules are considered as favorable prognostic factors for EC. The purpose of our study is to evaluate the prognostic potential of mir-375, 133, 143, 145 in primary EC, we summarized all the results from available studies, aiming delineating the prognostic role of mir in EC. Relevant studies were identified by searching databases including Medline, Embase, Web of science, Cochrane Library. The studies which explored the prognostic value of mir-375, 133, 143, 145 expressions on survival outcomes in patients with EC were included in this study. The hazard ratios (HR) and their responding 95% confidence interval (CI) were also extracted. A total of 25 studies were collected, including 1260 patients, and the prognostic values of four mirs in EC were analyzed. Survival outcomes including overall survival (OS), progression-free survival (PFS) and disease-free survival (DFS) were used as the primary endpoint to evaluate the prognostic value of mir. The pooled analysis results showed that up-regulation of mir-375 indicated favorable OS (HR=0.50; 95%CI: 0.37-0.69; *P*<0.001). In addition, the up-regulation of mir-133 (HR=0.40, 95%CI: 0.24-0.65, *P*<0.001), 143 (HR=0.40, 95%CI: 0.21-0.76, *P* < 0.001) and 145 (HR=0.55, 95%CI: 0.34-0.90, *P*<0.001) are also proved as protected factors in EC. Therefore, our study demonstrated that these mirs may have the potential to be used as prognostic biomarkers for EC in clinical practice.

## Introduction

Esophageal carcinoma (EC) is a common and fatal gastrointestinal malignant tumor which is a group of heterogeneous malignancies including squamous cell carcinoma (ESCC) and adenocarcinoma (EAC). Its clinical characters are associated with races, geographical distribution and other risk factors. EC is currently the sixth most common cancer worldwide (1), and is also one of the most deadly malignant tumor (2). According to the latest research, in 2020, there were 604000 cases of EC and 544000 deaths worldwide (3). Since the symptoms of early stage in EC are easy to be neglected, patients with EC are often diagnosed at late stage, and the optimum opportunity for surgery is lost (4). As a result, the five-year survival rate of EC patients is low, only about 30% (5). Although the advancement in therapeutic technologies including surgery, chemotherapy, radiotherapy and other novel treatments for EC has been greatly upgraded (6), it is still essential to find new biological markers to assess the prognosis of EC patients after surgery (7).

Non-coding RNA plays a vital role in cancer biology and provides potential targets for cancer intervention (8, 9). Early studies had indicated that non-coding RNA had the potential clinical utility for diagnosing and prognosticating cancers (10). One type of non-coding RNA is called micro-RNA (mir), which ranges in size from 20-24 nucleotides (11). In recent years, many studies have confirmed that mirs regulate gene expression by targeting mRNA for translational inhibition or cleavage, thereby involving in various biological phenotypes such as cell proliferation, differentiation, and apoptosis (11–13). Some mirs are considered to be related to the occurrence and development of tumors. Meanwhile, some of them were used as biomarkers for cancer detection and prognosis (14, 15). Mir-375 has been confirmed by studies that it is widely present in various tissues, which has been characterized as an important cancer-related mir (16, 17). The expression of mir-375 has been proved significantly reduced in malignant tumor cells (18). Several studies had reported that mir-375 were related to the prognosis of EC with low-expression in cancer tissue (19, 20), but nowadays, some more new researches were conducted to detect the mechanism of mir-375 may involve in EC, especially in prognosis, we collected entire of these data to make an update. On the other side, no published meta-analysis had investigated the impact of mir-133, 143, 145 on EC prognosis. In order to comprehensively and systematically investigate these mirs in EC prognosis, we conducted this meta-analysis to investigate the prognostic value of mir-375, 133, 143 and 145 while discussing the therapeutic potentialities of these mirs.

## Materials and methods

### Literature retrieval strategy

In order to obtain potentially eligible documents, we carefully searched the Medline, Embase, Web of science, Cochrane Library databases using the following search strategies and terms: (((((((esophageal cancer [Title/Abstract]) OR esophageal carcinoma [Title/Abstract]) OR esophageal squamous cell carcinoma [Title/Abstract]) OR esophageal esophageal adenocarcinoma [Title/Abstract])) AND (((MicroRNA [Title/Abstract]) OR miRNAs[Title/Abstract]) OR RNA, Micro [Title/Abstract])) AND (((prognostic [Title/Abstract]) OR prognosis [Title/Abstract]) OR survival [Title/Abstract])) updated until July 1, 2022. The retrieved related reviews and meta-analysis lists are also manually checked to identify more relevant information.

### Inclusion and exclusion criteria

The eligible studies included in this meta-analysis meet the following criteria (1): studies have reported specific methods of collecting mirs through surgical resection or blood collection and measuring their expression (2); the patients in studies had received treatment options such as surgery, radiotherapy or chemotherapy; (3) studies clearly illustrate the correlation between mir expression and survival outcomes of patients such as overall survival (OS), disease-free survival (DFS), progression-free survival (PFS), et al; (4) the patients were grouped according to the level of mir expression; (5) sufficient data are reported, and the hazard ratio (HR) and 95% confidence interval (CI) of mir expression can be retrieved based on the data in the article. The exclusion criteria are: (1) non-esophageal carcinoma patients; (2) duplicated studies; (3) studies using only *in vitro* cell lines; (4) animal experiments; (5) reviews, letters, case reports and expert opinions; (6) data could not be extracted or original data was lost.

### Data extraction and quality assessment

All eligible research data and information are independently extracted by two independent researchers (Fang and Zhou). The collected data information of various studies including: author, publication year, race, cancer type, patient number, sample type, mir detection method, survival outcomes, cut-off value and its 95% confidence interval. Based on the study population, intervention, comparison and outcome (PICO) format, the PICO of this study is defined bellow: P: patients with EC; I: patients with high mirs expression level; C: patients with low mirs expression level; O: survival outcomes. To reduce the bias of data extraction, the survival curve data was evaluated by other independent individual (Li), and the Engauge Digitizer version 4.1 software was used to analyze the survival curve. The original data and additional information needed for this study were obtained by contacting the authors of the included studies. The Newcastle-Ottawa Scale (NOS) was applied to assess the quality of all included studies (S1). The NOS scores including selection, comparability, and outcome, the score ≥6 was considered as study with high quality (21).

### Statistical analysis

The high or low expression of mir is defined according to the cut-off value provided by the author. The pooled HR and the corresponding 95% confidence interval were used to assess the correlation between mir expression and prognosis. All statistical analyses of this Meta-analysis are performed using Stata12.0 software (StataCorp, College Station, TX, USA) in accordance with PRISMA (S2) guidelines. The heterogeneity among the included studies was determined by the chi-square-based Q test and the I^2^ statistics; the *P* value of the Q test was ≤0.05; the I^2^ value ≥50% considered that the study had significant heterogeneity, the random-effects model was used to pool the effect size (*P*_Q_ ≤ 0.05, I^2^≥50%). If the *P* value of the Q test was >0.05; the I^2^ value <50%, we considered that the research heterogeneity is acceptable (*P*_Q_>0.05, I^2^<50%), and the fixed-effects model was used to analysis. To reduce the heterogeneity among studies and understand the prognostic value of mir-375, basing on multiple criteria we conducted a subgroup analysis of cancer type, sample type, race. To test the reliability of the main outcomes in our analysis, we conducted a sensitivity analysis of the included studies by removing one single study in turn and estimated whether there was publication bias among the studies using Begg’s test by assessing the asymmetry of an inverted funnel plot.

## Results

**Figure 1:**
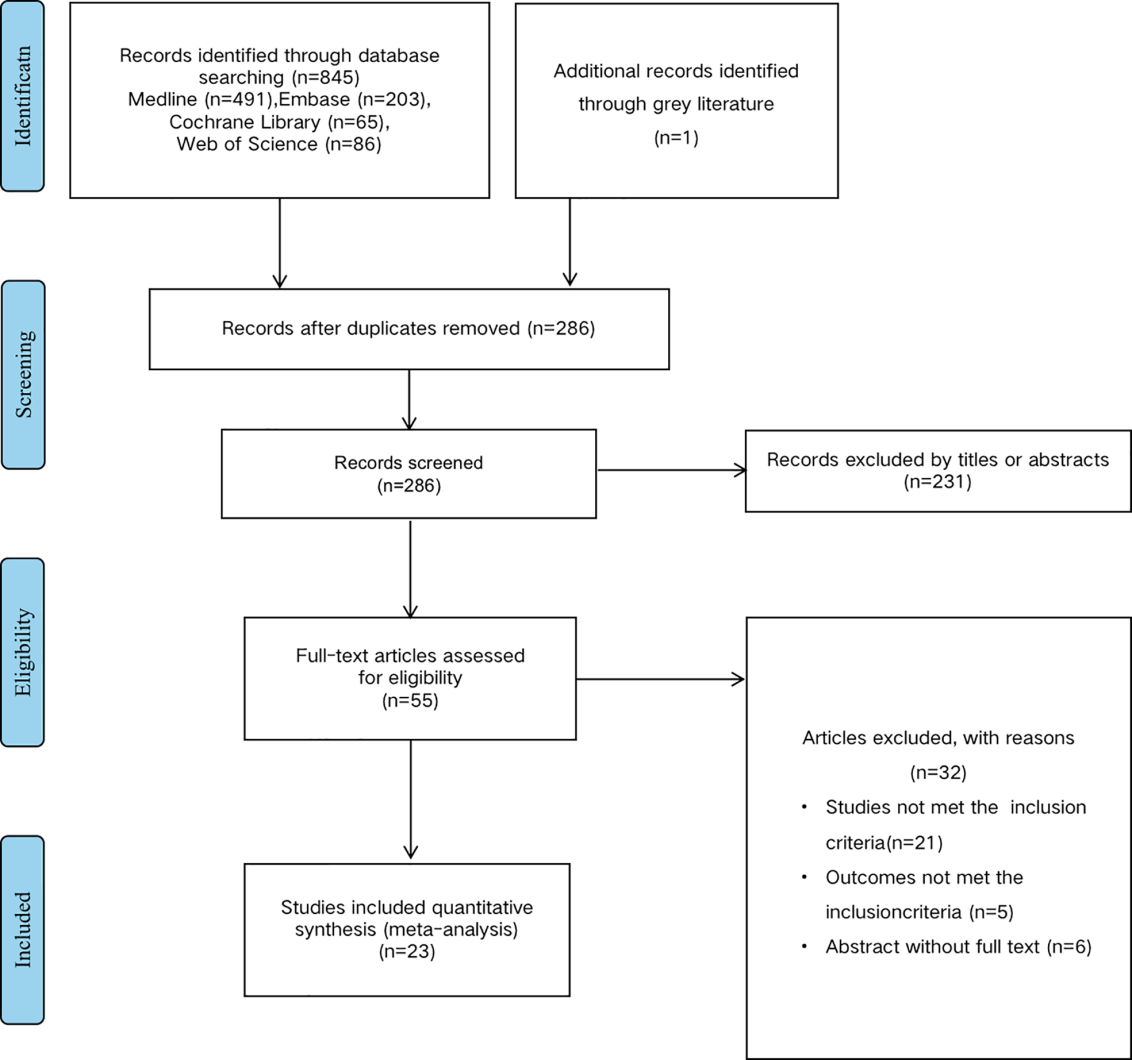
Methodological flow chart of the review.

According to the mentioned criteria above, we have identified 25 studies from the preliminary literature search from databases shown in the flow diagram (**Figure 1**). Exclude duplicate documents 560 articles. After screening the research title and abstract, 231 articles that are not related to the research content are excluded. We read the full text of 55 articles, and further excluded 32 articles because relevant data could not be extracted or couldn’t meet the inclusion criteria. A total of 25 published studies met the inclusion criteria for our meta-analysis which had analyzed the correlation between mir and EC survival outcomes. There are a total of 13 studies reported the prognosis of mir-375 and EC (16, 22–31), 12 articles reported mir-133 (32–34), mir-143 (35–37) and mir-145 (38–43), and all of these studies discussed the prognosis of EC. The total number of patients included in the study was 1260, ranging from 22-249. The category of cancer is ESCC or EAC and quantitative real-time PCR (qRT-PCR), microarray et al, were used to detect the expression of mir in tissue or blood. The cut-off values of mir expression varied in different studies, 20 studies reported the cut-off value of mir, including the mean, median, percentile, and fixed value. For mir-375, 9 studies were conducted in Asian populations, and 4 were conducted in Western. The study populations of mir-143 and mir-133 were only Asian race, as for mir-145, both patients in Asia and Western countries were included. More detailed information is summarized in (**Table 1**).

**Table 1:** The main characteristics of all included studies in this meta-analysis.

Micro-RNA	Name	Year	Population	Sample	Cancer type	Treatment	N	HR source	Cut-off	Age	Method	RESULT	NOS score
Mir-375	Mathé	2009	American	Tissue	ESCC	Surgery and chemoradiation	70	Multi	Median	<62: 28; ≥62: 42	Microarray and qRT-PCR	OS	6
	Mathé	2009	American	Tissue	EAC	Surgery and chemoradiation	62	Multi	Median	NA	Microarray and qRT-PCR	OS	6
	Li	2013	Chinese	Tissue	ESCC	Surgery	249	NA	NA	<60: 105; ≥60: 144	ISH	OS	7
	Wu	2014	Chinese	Tissue	ESCC	Surgery	194	Multi	Mean	<60: 106; ≥60: 88	qRT-PCR	OS	7
	Lv	2016	Chinese	Plasma	ESCC	Surgery	126	NA	Median	Mean 59.3	Microarray	OS	7
	Hu	2016	Chinese	Tissue	EC	Surgery	88	Multi	Mean	≥65: 57; <65: 31	qRT-PCR	OS	6
	Winther	2015	Danish	Tissue	ESCC	nCRT and surgery	129	NA	Median	36–81, mean 63	qRT-PCR	OS	7
	Winther	2015	Danish	Tissue	EAC	nCRT and surgery	66	NA	Median	32–86, mean 64	qRT-PCR	OS	7
	Xu	2019	Chinese	Tissue	ESCC	Surgery	43	Multi	The 75th percentile	41-79	qRT-PCR	OS	6
	Kong	2011	Chinese	Tissue	ESCC	Surgery	60	NA	NA	≤66:23; >66: 37	qRT-PCR	OS	6
	Kong (1)	2011	Chinese	Tissue	ESCC	Surgery	60	NA	NA	≤66:23; >66: 37	qRT-PCR	PFS	6
	Shuhei	2012	Japanese	Plasma	ESCC	Surgery	50	Multi	ROC curves	≤65: 25; >65: 25	qRT-PCR	OS	7
	Yuka	2015	Japanese	Tissue	ESCC	Surgery	85	NA	NA	≤65:37; >65: 48	qRT-PCR	OS	7
	Li	2015	Chinese	Plasma	ESCC	Radiotherapy and surgery	38	NA	Median	<65: 17; ≥65: 21	qRT-PCR	OS	6
	Li (1)	2015	Chinese	Plasma	ESCC	Radiotherapy and surgery	38	NA	Median	<65: 17; ≥65: 21	qRT-PCR	PFS	6
Mir-133	Lin	2017	Chinese	Tissue	ESCC	NA	58	Multi	NA	<65: 34; ≥65: 24	ISH	OS	6
	Gao	2016	Chinese	Tissue	ESCC	Surgery	126	Multi	Median	<55: 57; ≥55: 69	qRT-PCR	OS	7
	Akanuma	2014	Japanese	Tissue	ESCC	Surgery	140	Uni	Normal pair	<65: 81; ≥65: 59	qRT-PCR	OS	7
Mir-143	Liu	2019	Chinese	Tissue	ESCC	Surgery	44	Uni	Normal pair	<50: 19; ≥50: 25	qRT-PCR	OS	6
	He	2016	Chinese	Tissue	ESCC	Surgery	80	Multi	50-fold change	Mean 60	qRT-PCR	OS	6
	Zhang	2016	Chinese	Tissue	ESCC	Chemotherapy and surgery	31	Uni	NA	18–75	qRT-PCR	OS	6
Mir-145	Tanaka	2013	Japanese	Tissue	ESCC	nCRT and surgery	64	Uni	Median	45–80, median 67.5	qRT-PCR	DFS	6
	Augustine	2012	Canadian	Tissue	EC	Surgery and chemoradiation	25	NA	Median	NA	Microarray	DFS	6
	Feber	2011	British	Tissue	EAC	Surgery	45	Uni	Median	NA	Microarray	OS	6
	Jin	2019	Chinese	Tissue	ESCC	Surgery	126	Multi	Median	≥60, 57; <60, 69	qRT-PCR	OS	7
	Shimonosono	2018	Japanese	Tissue	ESCC	Surgery	22	NA	Median	52-84	qRT-PCR	OS	6
	Hamano	2015	Japanese	Tissue	EC	Surgery and chemotherapy	98	Multi	Median	63.2 ± 8.5	qRT-PCR	OS	7

Tissue, including patients’samples from frozen tissues and formalin-fixed paraffin-embedded; ESCC, esophageal squamous cell cancer; EAC, esophageal adenocarcinoma; EC, esophageal carcinoma; nCRT, neoadjuvant chemoradiation therapy; ISH, in situ hybridization; qRT-PCR, quantitative real-time PCR; NA, information not afforded; OS, overall survival; DFS, disease-free survival; PFS, progression-free survival; (1), the same study conducted by same author, and the author use PFS as the survival outcome to analyze.

**Figure 2:**
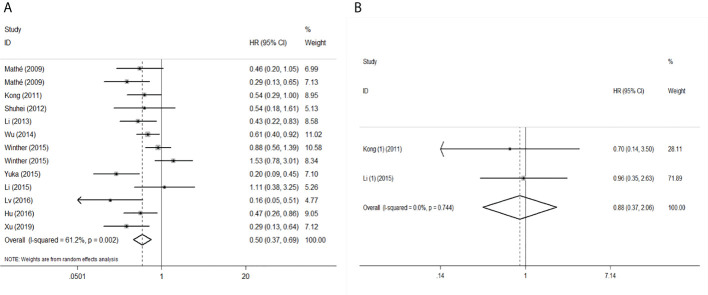
Forest plot of pooled HR of mir-375 in predicting survival outcomes in EC. **(A)** mir-375 and OS. **(B)** mir-375 and PFS.

**Figure 3:**
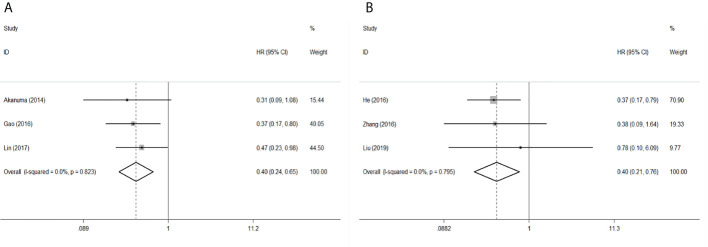
Forest plot of pooled HR of mir-133 and mir-143 in predicting survival outcomes OS in EC. **(A)** mir-133. **(B)** mir-143.

**Figure 4:**
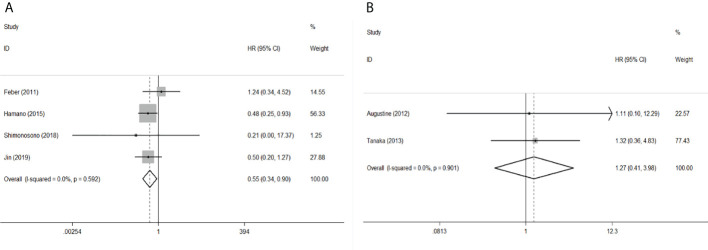
Forest plot of pooled HR of mir-145 in predicting survival outcomes in EC. **(A)** mir-145 and OS. **(B)** mir-145 and DFS.

**Figure 5:**
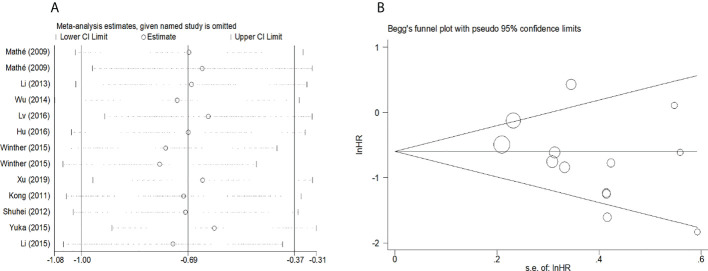
**(A)** Sensitivity analysis for meta-analysis of mir-375. **(B)**Funnel plots of publication bias for meta-analysis of mir-375.

To evaluate the OS for mir-375, a random-effects model was applied since there was heterogeneity among (I^2^ = 61.2%, *P*=0.002), pooled HR was 0.50 (95%CI: 0.37-0.69, *P*<0.001), in addition, the high expression of mir-375 showed no correlation with PFS the pooled HR was 0.88 (95%CI:0.37-2.06, *P*>0.05) (**Figure 2**), indicating that up-regulated mir-375 may be associated with better OS in EC. As for mir-143 (I^2^ = 0.0%, *P*=0.795) and mir-133 (I^2^ = 0.0%, *P*=0.823) studies evaluating OS were of no statistical heterogeneity, we use a fixed model to pool the HR. The results showed that up-regulated mir-143 and mir-133 was significantly associated with good OS outcome in ESCC with the pooled HR were 0.40 (95%CI: 0.21-0.76, *P*<0.001) and 0.40 (95%CI: 0.24-0.65, *P*<0.001) (**Figure 3**). On the other hand, to evaluate the prognosis of mir-145, we use fixed model to pool the HR, because there were no statistical heterogeneity found in the OS (I^2^ = 0.0%, *P*=0.592) and DFS (I^2^ = 0.0%, *P*=0.901) of mir-145, the results showed that high expression of mir-145 was associated with good OS in EC pooled HR was 0.55 (95%CI:0.34-0.90, *P*<0.001) but no correction was found in DFS because the pooled HR was 1.27 (95%CI: 0.41-3.98, *P*>0.05) (**Figure 4**). As shown in the figure, we have performed a sensitivity analysis picture of mir-375 to see if a single study could have significant impact on the pooled HR for survival, the results were not significant altered by removing anyone of the included studies, and Begg’s funnel chart (**Figure 5**) shows that no significant publication bias has been found (*P*=0.142). Based on the NOS assessment, the scores of these studies were from 6 to 7, indicating that the quality of the included studies is high, therefor all studies could be used in the subsequent analysis.

**Figure 6:**
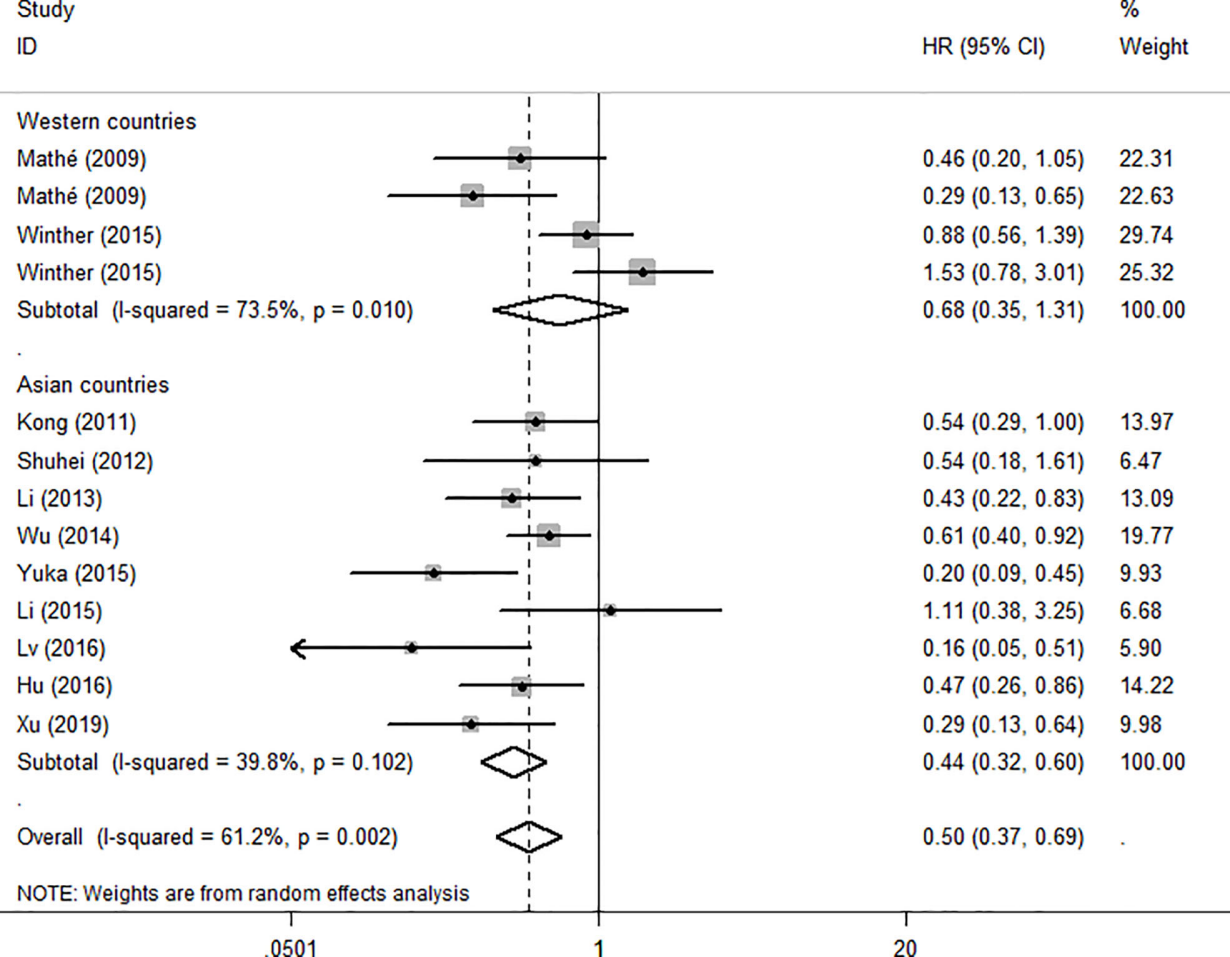
Forest plot showing subgroup analysis of the selected studies about the prognostic significance of mir-375 in patients with different races.

**Figure 7:**
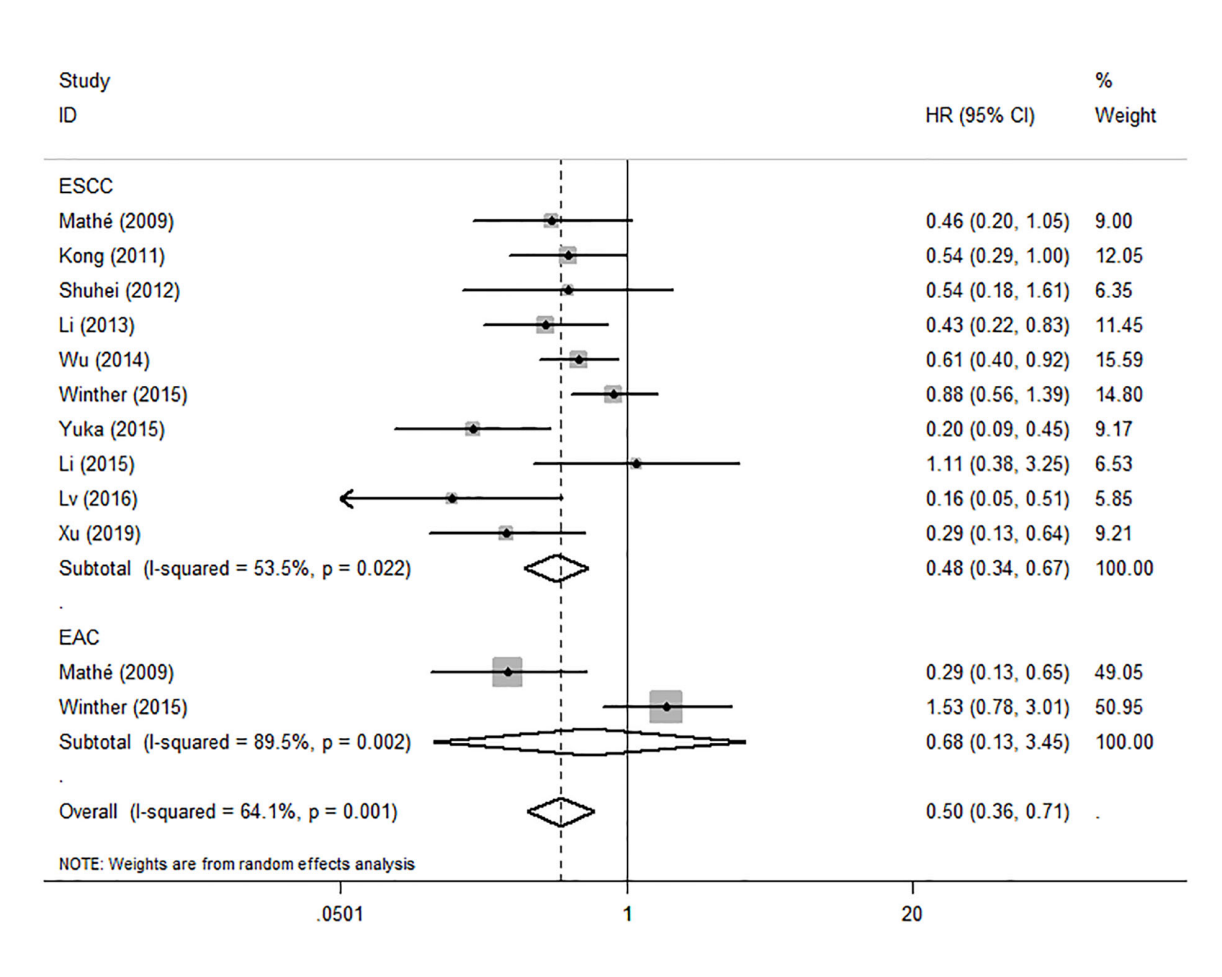
Forest plot showing subgroup analysis of the selected studies about the prognostic significance of mir-375 in patients with different cancer types.

**Figure 8:**
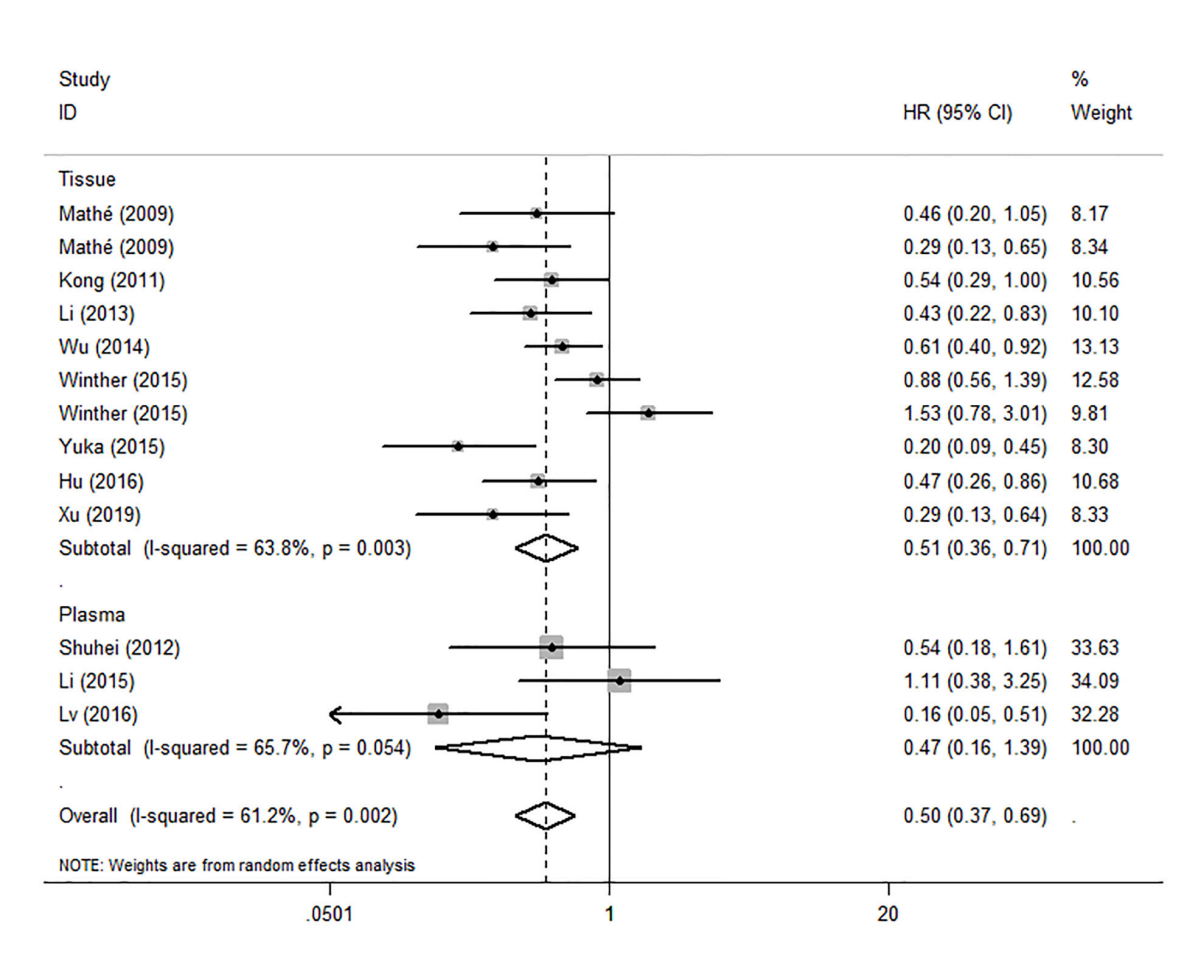
Forest plot showing subgroup analysis of the selected studies about the prognostic significance of mir-375 in patients with different sample types.

Because the heterogeneity among the effect size of mir-375, we conducted a subgroup analysis to find more information. The subgroups were stratified based on following criteria: cancer type, sample type, race. In the subgroup of patient race, we found that up-regulation of mir-375 significantly related to good prognosis in Asian patients (HR: 0.44, 95%CI: 0.32-0.60, *P*<0.01; random) but not in western patients (HR: 0.68, 95%CI: 0.35-1.31, *P*>0.05; random) and there was heterogeneity in the data, so we use random-effects model to analysis (**Figure 6**). In cancer type subgroup, higher mir-375 expression related to good prognosis in ESCC (HR:0.48, 95%CI: 0.34-0.67, *P*<0.01; random), no correlation was found in patients with EAC (HR: 0.68, 95%CI: 0.13-3.45, *P*>0.05; random) (**Figure 7**). The association between higher mir-375 expression and good OS outcomes was statistically significant in sample type from patients’ tissue (HR: 0.51, 95%CI: 0.36-0.71, *P*<0.01; random) but not from plasma (HR: 0.47, 95%CI: 0.16-1.39, *P*>0.05; random) (**Figure 8**). The pooled HR value and their 95%CI in each subgroup are demonstrated in figures above. By conducting such subgroup analysis could we find the impact of higher mir-375 expression on prognosis of EC patients with different clinical characteristics.

## Discussion

In recent years, various mirs have been confirmed to exhibit prognostic value in a variety of human cancers, and a great many of mirs have also been proved acting as a co-effector with other elements in tumor progression such as: cellular proliferation, survival, migration, and immune evasion. Thus, the mirs play an important role in the field of malignant tumor (10). The study by Zhao et al (44), showed that mir-375 not only reduced the stemness, but also decreased adriamycin resistance of breast cancer cells, indicating that mir-375 may inhibit the stemness breast cancer cells by targeting JAK2. Xie et al (45), have proved that low expression of mir-375 are significantly correlated with the occurrence and development of liver cancer. Furthermore, Zhang et al (46), found that mir-133 could inhibit the growth of triple-negative breast cancer by targeting YES1. In general, mir-143 and 145 were also clarified that they played a crucial role in suppressing tumor developing in other human cancer (47, 48). Beyond that, Yi et al (49), proved in their study that mir-375 could suppresses ESCC cells invasion and metastasis by direct targeting of SHOX2 and the interaction of mir-375 and SHOX2 could still affect EMT phenotypes. In addition, the down-regulated level of mir-375 was detected in primary ESCC, and was significantly associated with advanced stage and distant metastasis. The results of the study conducted by Osako et al (50), showed that mir-375 markedly inhibits cancer cell migration and invasion by regulation of MMP13 in ESCC, demonstrating that mir-375 may involve in MMP13 axis and affect the occurrence and development of EC.

Mirs played a key role in tumor pathology, that might partly clarify the association between their expression and EC prognosis and made mirs become a hotspot in oncology researching field. Considering the high mortality rates of EC may partly due to the lack of accurate prognostic biomarkers, in recent years, growing number of researchers had investigated the prognostic value of mirs in EC. Gao et al (51), conducted a meta-analysis focusing on mirs affecting EC prognosis. In their study, the number of literatures investigating these mirs was only 12 which was dated up to 2018. In our study, we made an upgradation of data focusing on mir-375,133,143 and 145 (the specific number of included studies is double to Gao’s) published up to November 20, 2021. Importantly, we included 15 studies which investigated the correlation between mir-375 expression and clinical survival outcomes of EC patients. With sufficient studies amount, we could achieve subgroup analysis to comprehensively and systematically investigate these mirs in EC prognosis and to make the conclusion more convincing. Up-regulations of mir-375,133,143 and 145 are associated with favorable prognosis. In addition, according to recent reports, mir was also used as a potential therapeutic targeted molecule in tumor. Many researches had tested the mechanism mirs involving in therapy to anti various human cancers. A study conducted by Campayo showed that up-regulation of mir-375 can increase the sensitivity of rectal cancer cells to chemoradiotherapy (52). On the other hand, Xu et al (53), showed mir-375 could enhance chemosensitivity to 5-fluorouracil in colorectal cancer. Fu et al (54), demonstrated that mir-375 could degrade HOXB3 and inhibit tamoxifen resistance in human breast cancer. Furthermore, Weidle et al (55), showed in their study that mir-133 had a negative impact on regulating cell-cycle and apotosis in gastric carcinoma. Záveský et al (56), illustrated that mir-143 were capable to inhibit the proliferation of ovarian cancer cells by *in vitro* experiment. Liu et al (57), found that up-regulation of mir-145 could perform as a tumor suppressor inhibiting the growth of non-small-cell lung cancer by targeting RIOK2 and NOB1. Nowadays, molecular-targeted therapy is becoming a vital treatment for malignant tumors and has attracted comprehensive attention worldwide. Therefore, these mirs may also have the potential to be used as targeted molecules for EC treatment in clinic practice.

Although the prognostic value of mir-375 was proved by our study statistically, it should be prudently comprehended because of the following reasons. First, according to the results of subgroups analysis, the pooled HR value showed no significant correlation in EAC which indicated that in different EC types mir-375 may involve in different mechanism which are still unclear to us. Similarly, Mathe et al,[14] also found down-regulation of mir-375 significantly associated with poor EC prognosis only in EAC patients with Barrett’ s esophagus. Considering of such discrepancy, more well-designed clinical researches with EAC samples should be conducted in the future to elucidate the relationship from different cancer types. Second, subgroups analysis also showed that sample types also affected a lot, when sample from plasma, the pooled HR showed no association in mir-375 expression and EC prognosis, contradicting to the sample from tissue which indicated the blood mir-375 may not be used as biomarker to detect the tumorigenesis and monitor tumor recurrence. Currently, mir-375 from tissues were more to be used to study. However, circulating biomarkers were more likely to be applied in clinic because they could be easily got and could be monitored through the whole progression of disease. Therefore, deeper clinical studies should be completed to verify whether there is a correlation between mir-375 expression both in tissue and blood. Third, in different races subgroups, up-regulation of mir-375 wasn’t related to good prognosis in western patients, this may because of the sample sizes from western countries were small and the conclusion might be biased. Fourth, previous studies had investigated the mirs overexpressed in EC patients. Therefore, in this study, we mainly focused on those mirs down-regulated in EC. According to the pooled HRs results, the included four mirs all act as protective factors, and the result might be different from the reality to a certain extent.

Though our meta-analysis proved that mir-375 has an association on the prognosis of patients with EC, there are still some limitations in this study. First, EC is a series of malignant tumor, ESCC and EAC consisted of the most common histological subtypes, but some of studies included in our research did not strictly distinguish the subtypes of EC, leading to a bias in the classification of cancer types. Second, most researchers have used the median or average as the cut-off value in their studies, lacking uniform standards for the cut-off value of mir expression in different studies, the pooled survival outcomes may deviate from the true value. Third, some HR values in this study were calculated by using software Engauge Digitizer to extract data from the survival curve which may bring minor deviations to the outcome. Fourth, there is significant methodological heterogeneity in clinical and baseline demographic characteristics, especially tumor stage, treatment methods which are most likely to affect prognosis in patents. Detailed staging statistics are not provided on patients in the included studies, and many studies have not described the treatment of patients with EC in detail, so subgroup analysis cannot fully reflect the sources of heterogeneity between studies and may lead to bias in patients’ homogeneity. Finally, our meta-analysis only collected perspective mirs evidence from databases but have no specific basic research to verify the exact mechanism mirs involving in EC, so the conclusion was less convincing. Hence, more necessary basic research based on EC patients’ tissues and cells should be completed to obtain accurate results and conclusions.

## Conclusion

The results of this meta-analysis show that high expression of mir-375, mir-143, mir-133 and mir-145 is significantly associated with a better prognosis in EC, indicating that these mirs could be used as prognostic factors for EC. Meanwhile, the recurrence of EC whether could be early detected by monitoring these mirs are still unknown. Furthermore, these novel mirs may also be utilized as potential therapeutic targets for EC treatment. For ESCC in Asian population, high expression of mir-375 is a significantly favorable factor, implying a potential therapeutic target for ESCC. Therefore, more high quality clinical researches are needed to investigate the prognostic and therapeutic value of mir-375.

## Data availability statement

The original contributions presented in the study are included in the article/**Supplementary Material**. Further inquiries can be directed to the corresponding author.

## Author contributions

YYu conceptualized the study, revise and proof the manuscript. PF, JZ and XL conceptualized the study and drafted the manuscript. SL, XX, QS, HZ, YYa and XZ collected the literature. All authors contributed to the manuscript revision and read and approved the submitted version.

## Funding

This study was supported by the National Natural Science Foundation of China (Grant No. 81970481)

## Conflict of interest

The authors declare that the research was conducted in the absence of any commercial or financial relationships that could be construed as a potential conflict of interest.

## Publisher’s note

All claims expressed in this article are solely those of the authors and do not necessarily represent those of their affiliated organizations, or those of the publisher, the editors and the reviewers. Any product that may be evaluated in this article, or claim that may be made by its manufacturer, is not guaranteed or endorsed by the publisher.
